# The role of laser and energy-assisted drug delivery in the treatment of alopecia

**DOI:** 10.1007/s10103-024-04015-0

**Published:** 2024-02-21

**Authors:** Eliza Balazic, Ahava Muskat, Yana Kost, Joel L. Cohen, Kseniya Kobets

**Affiliations:** 1 Division of Dermatology, Albert Einstein College of Medicine, 1300 Morris Park Ave., Bronx, NY 10461 USA; 2 AboutSkin Dermatology & AboutSkin Research, Greenwood Village, CO USA

**Keywords:** Laser, Energy-based devices, Ablative laser, Non-ablative laser, Radiofrequency microneedling, Alopecia, Androgenetic alopecia, Alopecia areata, Minoxidil, Triamcinolone, Patterned hair loss

## Abstract

It has been recently established that laser treatment can be combined with topical or intralesional medications to enhance the delivery of drugs and improve overall results in a variety of different dermatological disorders. The aim of this review is to evaluate the use of laser and energy-assisted drug delivery (LEADD) for the treatment of alopecia with a specific focus on ablative fractional lasers (AFL), non-ablative fractional lasers (NAFL), and radiofrequency microneedling (RFMN). A comprehensive PubMed search was performed in December 2022 for “laser-assisted drug delivery” as well as “laser” and “alopecia.” The evidence regarding LEADD for alopecia treatment is limited to two specific alopecia subtypes: alopecia areata (AA) and androgenetic alopecia (AGA)/pattern hair loss (PHL). LEADD with minoxidil and platelet-rich plasma (PRP) were evaluated for efficacy in both treatments of AA and AGA. LEADD with topical corticosteroids and intralesional methotrexate were studied for the treatment of AA, while LEADD with growth factors and stem cells were studied for the treatment of AGA. Multiple RCTs evaluated LEADD for topical corticosteroids with ablative fractional lasers for the treatment of AA. There is evidence in the literature that supports the use of topical minoxidil in combination with all devices for the treatment of AGA/PHL. All the reviewed studies show a positive treatment effect with LADD; however, some trials did not find LEADD to be superior to monotherapy or microneedling-assisted drug delivery. LEADD is a rapidly emerging treatment modality for the treatment of AGA and AA.

## Introduction

It has been recently established that laser treatment can be combined with topical or intralesional medications to enhance the delivery of drugs and improve overall results in a variety of different dermatological disorders [1]. This treatment is often referred to as laser-assisted drug delivery (LADD). However, there are other devices that can be used to enhance drug delivery like radiofrequency microneedling (RFMN). We propose a new term—laser and energy-assisted drug delivery (LEADD)—to include other forms of energy-based devices not included in LADD.

There are many types of alopecia with different treatment algorithms, but most rely on topical medications to the scalp. These topical medications such as topical minoxidil and triamcinolone acetonide (TAC) have been used with laser and energy-based devices for LEADD treatment for alopecia. The aim of this review is to evaluate the use of LEADD for the treatment of alopecia with a specific focus on ablative fractional lasers (AFL), non-ablative lasers (NAFL), and RFMN.

## Methods

A comprehensive PubMed search was performed in December 2022 for “laser-assisted drug delivery” as well as “laser” and “alopecia.” Articles were then screened for eligibility with the inclusion criteria as the article focused on LEADD for alopecia treatment, focused on human subjects, and was in English. The results were further refined to only include articles involving AFLs, NAFLs, and RFMN. Review articles were excluded. Ultimately, 18 articles were included in this review spanning from 2018 to 2022. Articles were evaluated for laser type and setting, drug, study design, selected outcomes, and study type. Randomized controlled trials (RCTs) were assessed for quality by the Jadad scale which is a five-point scale that addresses randomization (2 points), blinding (2 points), and accounting for all subjects including dropouts (1 point) [2].

## Results

Currently, the evidence regarding LEADD for alopecia treatment is limited to two specific alopecia subtypes: alopecia areata (AA) and androgenetic alopecia (AGA)/pattern hair loss (PHL) (Table 1).

**Table 1:** Study information including alopecia, drug, laser, settings used, study design and type, adverse events and selected outcomes

Type of alopecia	Drugs	Laser type	Settings	Study design	Selected outcomes	Adverse effects	Study type/Jadad Scale (max 5)	Reference
AA	Minoxidil 5%	NAFL (1550 nm Er:Glass; GSD, Shenzhen, China)	Energy: 10–15 mJ Intensity: 300 spots/cm^2^	10 treatments every 2 weeks with twice daily minoxidil until last laser treatment on 8 patients	3-point scale: Score 0 (no effect): 2 patients Score 1 (hair regrowth < 50% lesions): 1 patient Score 2 (hair regrowth > 50 lesions): 5 patients Relapse of one patient at 1 year follow-up	Mild erythema, mild broken hair shafts, and pain; number not reported	Case series/0	Wang et al. [3]
AGA	Minoxidil 5%	AFL (CO_2_; DEKA Smartxide2 DOT/RF c60, Italy)	Power: 5W Pulse energy: 51.6 mJ Density: 8.7% Fluence: 4.68 J/cm^2^ Spot size: 15 mm Dwell time: 500 μs	Three groups with 45 males total: Combined group: 6 sessions with 2-week intervals, followed immediately by topical minoxidil then twice daily Laser only: 6 sessions with 2-week intervals Minoxidil only: topical minoxidil twice daily for 3 months	Total hair count: significant baseline difference between the three groups. Significant post-treatment increase in all groups: Combined group (*p* = 0.001), laser (*p* = 0.005), and minoxidil group (*p* = 0.007) Thick hair thickness: no significant baseline differences between groups. Significant post-treatment increase only in combined group (*p* = 0.008) and laser (*p* = 0.042)	Erythema (33%), itching (16%) and post-inflammatory hyperpigmentation (7%)	Open-labeled non-randomized clinical study/0	Salah et al. [4]
AGA	Minoxidil 5%	NAFL (1550 nm Er:glass; Finescan, TNC Meditron, Bangkok, Thailand)	Energy: 6 mJ Density: 300 spot/cm^2^ Probe diameter: 7 mm	Randomized 30-person split scalp study for 24 weeks One half: laser one half of scalp at 2-week intervals for 12 sessions and minoxidil 5% twice daily Other half: minoxidil 5% twice daily alone	Difference in hair density and hair diameter: increased in combination group compared to monotherapy (*p* = 0.004, *p* = 0.034)	LEADD side: tolerable pain and warmth during procedure (9 patients), erythema (6), itchiness (4), and scaling (2). Topical: itching (5) and scaling (3)	Investigator blinded split scalp RCT/3	Suchonwanit et al. [5]
Pattern hair loss	Minoxidil 5%	FRMN (fractional radiofrequency microneedling; BodyTite, Derma Optic and Electronic Ltd, Chongqing, China)	Tip: 1 cm^2^ with 49 insulated 0.25-mm diameter microneedle electrodes Bipolar RF pulses: 1 MHz Power: 12 W Depth: 1.5 mm Pulse duration: 300 ms	Randomized 19-person split scalp study for 5 months One half: five FRMN treatments at 4-week intervals with topical minoxidil 5% twice daily Other half: topical minoxidil 5% twice daily	Mean change from baseline for mean hair count: increased in combined therapy group compared to monotherapy (*p* < 0.01) Difference in hair thickness after 5 months of treatment: increased in combined therapy group compared to monotherapy (*p* = 0.02)	LEADD: tolerable pain, pinpoint bleeding, erythema (all) Topical: dandruff(8)	Split scalp RCT/3	Yu et al. [6]
AGA	PRP (injected)	NAFL (Er:Glass)	Energy: 7 mJ Coverage: 9% Passes: 8	60 patients randomized study with treatments at 1 month intervals for 4 sessions Combination group: laser treatment then PRP injections Laser group: laser only PRP group: PRP only	No significant difference was found between groups Hair density: some improvements in 80% of combined group, 65% of laser group, and 70% of PRP group		RCT/2	Haddad et al. [7]
AGA	PRP	AFL (2940 nm Er:YAG; SP Dynamis, Fotona, Slovenia)	Fluence: 7.00 J/ cm^2^ Spot size: 7 mm Frequency 3.3 Hz	Retrospective study of 16 patients treated with laser monotherapy or combination therapy with PRP injections. Some patients were also on topical minoxidil and oral cosmeceuticals	No differences were found between different treatment groups. Most groups improved	No adverse reactions	Retrospective cohort clinical study/0	Day et al. [8]
AGA	PRP	AFL (CO_2_; Pentagon Grand, Daeju Meditech Engineering, Seoul, Korea)	Low-pulse: energy: 12 mJ Density: 800 spot/cm^2^ High-pulse: Energy: 22 mJ Density: 400 spot/cm^2^	Split-scalp (half-head) pilot study of 7 participants. Treatment every 2 weeks for 10 total treatments with 12-week follow-up One-half: high pulse energy followed by topical PRP Other half: low pulse energy followed by topical PRP	Mean total hair density: increased significantly in high pulse group compared to low pulse group (*p* = 0.023)	Tolerable pain (7), mild pruritis (2), dandruff (4)	Pilot study/0	Hanthavichai et al. [9]
AA	PRP, TAC (10 mg/mL)	AFL (10,600 nm CO_2_; Advanced Technology Laser Company, Ltd., Shanghai, China)	Power: 20 W Density: 4 pulses per inch Pulse duration/time: 3 ms	60 participants randomized with treatment every 3 weeks for four treatments with 4-week follow-up Group 1: laser and TAC Group 2: microneedling and TAC Group 3: laser and PRP Group 4: microneedling with PRP Groups 1 and 3: laser treatment was followed by application of topical drug Groups 2 and 4: drug was applied before, during, and after microneedling	Regrowth scales showed microneedling to be more effective than laser for drug delivery (*p* = 0.023) with TAC working better than PRP (*p* = 0.015). All treatment groups showed improvement	Laser: discomfort from heat in some patients Both groups: pain more tolerable than intralesional injections	RCT/3	El Mulla et al. [10]
AA	TAC (20 mg/ml)	AFL (CO_2_), RFMN (both devices not specified)	RFMN: Roller: 10-mm-width wheel with 6 coags/disc with 50 pins/coag Depth: 100–150 μm Diameter 80–120 μm Laser: Depth: 150–300 μm Diameter 125–150 μm	Case series of 5 patients treated with laser or RFMN then topical TAC then acoustic pressure wave ultrasound (US)	All participants had complete resolution of their lesions. Two patients with RFMN after three and six sessions, respectively. Two patients after laser treatment with laser resolving after one session. The fifth patient had laser treatment that did not follow the treatment steps	Mild burning sensation during procedure	Case series/0	Issa et al. [11]
AA	TAC (10 mg/mL)	AFL (CO_2_; Lutronics, Korea)	Tip: 120 μm Fluence: 50–60 mJ/cm^2^ Density: 100 microthermal zones (MTZ)/cm^2^	Case series with 8 patients with treatment resistant AA. Treatment consisted of laser, followed by TAC spray for 4–8 treatments	7 patients had excellent response (> 75% hair growth), 1 patient had “not good” response after 4 treatments	None reported	Case series/0	Majid et al. [12]
AA	TAC (10 mg/ml)	AFL (CO_2_; DEKA Smartxide, Italy)	Power: 7 W Pulse energy: 51.6 mJ Density: 8.7% Fluence: 4.687 J/cm^2^ Spot size: 15 mm Dwell time: 500 s (authors attempted unsuccessfully to contact authors to confirm	30 participants with treatment resistant AA randomized to LADD with TAC or microneedling with TAC with sessions every 3 weeks for 12 weeks	Treatment response at first follow-up (12 weeks) 13.3% in laser group and none in microneedling group. Black dot higher in microneedling group. No significant difference in effectiveness	Laser group: no significant adverse effects Pain score significantly less in laser group (*p* = 0.002)	RCT/1	Omar et al. [13]
AA	TAC (10 mg/ml)	AFL (CO_2_; Punto, DEKA, Italy)	Power: 10 W Dwell time: 500 ms, Stack: 2 Spacing: 700 m	30 participants randomized to monthly laser or microneedling, followed by TAC until resolution or for a maximum of 6 sessions	Both groups had a significant reduction in SALT score (*p* < 0.001) with reduction in SALT higher in microneedling group than laser group (*p* = 0.013)	No statistically significant difference in side effects between groups. Only mild pain and erythema were reported	RCT/2	Abd ElKawy et al. [14]
AA	Clobetasol propionate	AFL (ER: YAG; XS dynamics Fotona S1-121d, Ljubljana Slovenia)	Fluence: 3 J/cm^2^ Frequency: 3–5 Hz Mode: short pulse Spot size: 7 mm	30 subjects with AA had lesions randomized to LEADD or topical clobetasol alone. The laser treatment occurred every 2 weeks for 2–3 weeks, followed by one application of topical clobetasol. The other lesions were treated with daily clobetasol alone	Both groups showed significant improvement in SALT score with the combination therapy showing a greater effect (*p* = 0.035)	Laser group: pain and transient post-treatment erythema, edema, and pruritus	Comparative study/0	Shokeir et al. [15]
AA	Betamethasone	AFL (CO_2_; DEKA SmartXide, Italy)	Power: 16* Dwell time: 600 * Spacing: 600* Fluence: 2.13 J/cm^2^ *units not specified	30 patients received treatment for 4 months LEADD group: eight laser treatments every 2 weeks in addition to betamethasone cream after laser session and daily Laser group: eight laser treatments every 2 weeks Topical group: betamethasone cream daily	All groups showed statistically significant decrease in SALT score after treatment (all *p* = 0.005). Combined group reduced SALT compared to topical group (*p* = 0.003). Laser group also reduced SALT compared to topical group (*p* = 0.002). No difference between combined group and laser group was found	LEADD and laser group: discomfort during procedure and transient post-treatment scaling and erythema	Comparative study/0	Halim et al. [16]
AA	Methotrexate (intralesional)	AFL (CO_2_; CO2RE Candela, Massachusetts)	Fluence: 288 J/cm^2^ Coverage: 5% Passes: 2	Two cases treated with laser and intralesional methotrexate. Cases were additionally treated with pulse oral steroids	Case 1: hair regrowth with villous white hairs on dermoscopy at week 16 Case 2: repopulation by week 22	Transient pain, redness, mild transitory hyperpigmentation	Case series/0	Rodríguez-Villa Lario et al. [17]
AGA	Growth factors (GFs)	AFL (CO_2_; Pixel CO_2_, Alma Lasers Ltd., Esthetic Mode, Israel)	Tip: 50 mm Energy: 12–18 mJ/spot 361 spots/cm^2^ Density: 40%	27 participants were treated in this split scalp study with treatment sessions every 2 weeks for 6 total sessions with final evaluation 4 months after final treatment One half: laser followed by application of GFs using acoustic-pressure ultrasound. Then GFs were applied topically once every other day for 2 weeks Other half: during treatment session, application of GFs was done using acoustic-pressure ultrasound. Then GFs were applied topically once every other day for 2 weeks	Mean hair density increased significantly in both groups (*p* < 0.001). The mean change from baseline was also significantly higher in combined group (*p* = 0.003)	Post-treatment erythema (27), edema (7), pruritus (8), dryness (3), seborrheic dermatitis (2), and dandruff (7)	Split-scalp RCT/2	Huang et al. [18]
Pattern hair loss	GFs	NAFL (1927-nm-fractionated thulium laser; LASEMD, Lutronic Corporation, Goyang, Korea)	Power: 5 W Energy: 4 mJ or 6 mJ Pulse count: 100–140 pulses	10 participants treated in this split scalp study with 12 laser sessions at 1-week intervals with follow-up at 4 and 12 weeks after laser treatment Half scalp: laser treatment only Other half: laser treatment then topical GF solution	Hair counts and hair thickness significantly increased 1 week after final treatment compared to baseline (both p < 0.001) in both groups	No side effects reported	Split scalp RCT/2	Cho et al. [19]
AGA	Adipocyte-derived mesenchymal stem cell-conditioned media (ADSC-CM)	NAFL (Mosaic; Lutronic Corporation, Goyang, Korea)	Pulse: 5 mJ/ Spot density: 500 spots/cm^2^	30 participants were randomized and treated in this split-scalp study. The whole scalp was treated with ADSC-CM or placebo solution once per week with weekly at home microneedling. The scalp was treated with a single laser session at the initial visit	Hair density: ADSC-CM group significantly increased hair density compared to placebo (*p* < 0.05)	No adverse events reported	RCT/5	Lee et al. [20]

*AA* alopecia areata, *ADSC-CM* adipocyte-derived mesenchymal stem cells-conditioned media, *AFL* ablative fractional laser, *AGA* androgenetic alopecia, *GF* growth factors, *NAFL* non-ablative fractional laser, *PRP* platelet rich plasma, *RCT* randomized controlled trial, *TAC* triamcinolone acetonide

### Minoxidil

Minoxidil is a mainstay topical therapy for AGA. Several studies investigated the efficacy of topical minoxidil as a LEADD treatment. A split-scalp RCT examined monthly RFMN with twice daily topical 5% minoxidil vs. topical minoxidil only for PHL. The study found a significant increase in hair count (*p* < 0.01) and hair thickness (*p* = 0.02) in the LEADD side after 5 months [6]. Another split-scalp RCT examined LEAAD using bimonthly NAFL laser with twice daily topical 5% minoxidil vs. topical minoxidil alone for AGA. This study found increased hair density and hair diameter in the LEADD group compared to monotherapy (*p* = 0.001) [5]. An open-labeled non-randomized clinical study examined the use of AFL (CO_2_) for AGA with a laser only group receiving treatment every 2 weeks, a twice daily topical minoxidil group, and a combination group receiving the topical and laser treatments. After treatment, hair thickness increased significantly only in the LEADD (*p* = 0.001) and the laser only group (*p* = 0.001), while hair count increased significantly in all groups: LEADD group (*p* = 0.001), laser group (*p* = 0.005), and minoxidil group (*p* = 0.007) [4]. All groups saw significant improvement in the LEADD group; however, different laser and energy devices were used, making it difficult to draw larger conclusions on the best device type for LEADD with topical minoxidil for AGA/PHL.

While topical minoxidil is not traditionally used for the treatment of AA, one case series examined the use of LEADD with bimonthly NAFL and twice daily topical minoxidil 5% for 8 patients. Hair regrowth of greater than 50% of lesions was seen in five patients with one patient seeing no hair regrowth [3].

### Platelet rich plasma

A RCT with three groups—LEADD with monthly NAFL, followed by intralesional PRP, monthly NADL only, and intralesional PRP only—found no significant differences between groups with some improvements in 80% of LEADD group, 65% of laser group, and 70% of PRP group [7]. A pilot study examining LEADD with AFL (CO_2_), followed by intralesional PRP for AGA tested low vs. high pulse settings with the high pulse group showing significantly increased mean total hair density compared to low pulse group (*p* = 0.023) [9]. A retrospective study of patients treated with NAFL (Er:YAG) monotherapy or in combination with PRP at every other session. This study did not find a significant difference between groups, and all showed improvement; however, most patients were also on topical minoxidil and oral cosmeceuticals [8]. LEADD with PRP for AGA is an emerging therapy with currently limited evidence on whether it is superior to monotherapy.

### Topical corticosteroids

Topical and intralesional corticosteroids are used to treat AA but can be combined with laser or energy devices for combination therapy. A RCT assigned patients to monthly microneedling or AFL (CO_2_), both followed by application of triamcinolone acetonide (TAC) for six sessions or resolution of lesions. Both groups had a statistically significant reduction in severity of alopecia Tool (SALT) score (*p* < 0.001) with a significant difference between groups favoring the microneedling group (*p* = 0.013) [14]. Another comparative study for treatment resistant AA compared microneedling or AFL (CO_2_), both followed by application of triamcinolone acetonide (TAC). Both groups had significant improvement at each follow-up with the only significant difference between groups being the presence of black dot dermoscopy sign which was more present in the laser group (46.7 vs. 13.3%) [13]. Two small case series of LEADD with TAC for AA using AFL (CO_2_), and RFMN saw positive results [11, 12].

One RCT directly compared microneedling-assisted drug delivery and LEADD using PRP and TAC. The sixty-person study randomized participants to four groups: AFL (CO_2_) and TAC, microneedling and TAC, AFL (CO_2_) and PRP, and microneedling and PRP. Each group had treatments every 3 weeks for four treatments. All treatment groups showed improvement; however, regrowth scales showed microneedling to be more effective than laser for drug delivery (*p* = 0.023) with TAC working better than PRP (*p* = 0.015) [10]. LEADD with TAC is an effective treatment for AA, but it may not be superior to microneedling-assisted drug delivery.

Other topical corticosteroids have been used for LEADD in AA. A trial comparing AFL (Er:YAG) followed by clobetasol and topical clobetasol alone found significant improvement in SALT score in both groups with the combination therapy showing a greater effect [15]. Another study using betamethasone compared LEADD using AFL (CO_2_) with betamethasone, AFL (CO_2_) alone, and betamethasone alone. All groups showed statistically significant decrease in SALT score after treatment (all *p* = 0.005) with LEADD group and laser group reducing SALT compared to topical group (LEADD: *p* = 0.003; laser *p* = 0.002) [16].

### Other topicals: growth factors and methotrexate

A split-scalp RCT for AGA had one half of the scalp treated with AFL (CO_2_) with growth factors (GFs) applied to the full scalp every 2 weeks for 6 weeks. Mean hair density was significantly increased in both groups (*p* < 0.001) with a significant difference between groups favoring the LEADD group (*p* = 0.003) [18]. A similar split-scalp study for PHL performed weekly full-scalp NAFL (Thulium), followed by application of GFs to one half of the scalp. Both groups showed significantly increased hair counts (LEADD *p* = 0.001; laser *p* < 0.001) at 1 month post-final treatment session [19].

A split-scalp study for AGA investigated adipocyte-derived mesenchymal stem cell-conditioned media (ADSC-CM). The full scalp was treated with NAFL (Er:Glass) once followed by ADSC-CM to one half and placebo solution to the other. This study provided with an at-home microneedling device that participants used to the full scalp once a week along with weekly topical treatment application. The ADSC-CM group had significantly higher final densities compared to placebo (*p* < 0.05) [20]. The at-home microneedling device likely contributed more than the sole laser session in this study.

A case series of two patients with AA found a good response after treatment with AFL (CO_2_) and intralesional methotrexate (MTX). The first case saw regrowth of white hairs at 16 weeks after sessions of AFL followed by MTX every 2 weeks along with a pulse treatment of dexamethasone. The second case after failed therapy received AFL followed by MTX every 20 days along with 3-day prednisone treatment with complete response by week 22 [17]. While this case report provides limited evidence to the efficacy of AFL with MTX, it explores a new treatment option for patients who have failed intralesional and systemic corticosteroids.

## Discussion

LEADD for alopecia is an emerging field as 50% of the studies reviewed were published in 2022 reflecting a rapidly growing interest in treating alopecia with LEADD techniques. These studies only focus on treatment for AGA and AA with room for expansion into other types of alopecias in the future. All the studies noted in this review saw a positive treatment effect for the LEADD groups. The strongest evidence for LEADD in alopecia is for the use of AFL with topical corticosteroids. Minoxidil was combined with all devices, AFL, NAFL, and RFNM for a positive effect. The results were mixed on whether LEADD is superior to monotherapy or microneedling. Two of the RCTs for AGA comparing LEADD to microneedling-assisted drug delivery found the microneedling to be the superior treatment modality [10, 14]. Larger studies with different drugs are needed to directly compare treatment methods, various settings and depths of devices for treatment of the scalp.

Many articles compared the LEADD treatment to topical therapy only; however, three articles directly compared LEADD to device alone. All three studies did not find any difference between LEADD treatment and laser only. These studies investigated AFL and topical minoxidil 5% for AGA, NAFL, and injected PRP for AGA, and AFL and betamethasone for AA [4, 7, 16]. This may indicate that laser monotherapy may be an effective treatment for hair growth, as all studies showed improvement in alopecia.

It is important to note that the topical minoxidil studies may not be a true LEADD effect, as most studies had the participants continue to apply the minoxidil twice daily for the duration of the study. LADD works through fractional photothermolysis via a variety of mechanisms including dermal remodeling [21]. When using AFLs, channels are created within the skin to drive the drug delivery deeper; however, these channels close as reepithelization occurs which occurs up to 48 h after the AFL treatment [22, 23]. In these studies, the LEADD that occurs immediately post-treatment is synergistic with the daily application of the topical drug. This is relevant to clinical practice as patients with AGA participating in LEADD treatments should continue their topical regimens to achieve maximal results.

The LEADD was relatively well-tolerated in all studies with most studies reporting transient side effects related to laser procedures most commonly pain and erythema.

There is a lack of large, high-quality RCTs relating to LEADD treatment of alopecia which is evident by the low Jadad scores ascribed to most of the RCTs evaluated. It is difficult to conduct double-blinded studies, as it would be challenging to use a sham laser device. Only one of the articles utilized a topical placebo.

## Conclusion

LEADD is a rapidly emerging treatment modality for the treatment of AGA and AA. Traditional drug modalities can be combined with laser treatments for an augmented effect. Larger, well-designed studies are needed to draw more definitive conclusions.
